# Verification of the Cage Stability and the Superiority of Titanium Coating in the Bone Fusion of Transforaminal Lumbar Interbody Fusion Using Polyetheretherketone Cages

**DOI:** 10.7759/cureus.77485

**Published:** 2025-01-15

**Authors:** Kazutaka Masamoto, Shimei Tanida, Bungo Otsuki, Shunsuke Fujibayashi

**Affiliations:** 1 Spine Surgery, Shiga General Hospital, Moriyama, JPN; 2 Orthopaedics, Kyoto University Hospital, Kyoto, JPN; 3 Department of Orthopaedic Surgery, Yoshikawa Hospital, Kyoto, JPN

**Keywords:** fusion rate, propensity score, titanium-coated peek, tlif, vertebral endplate cyst

## Abstract

Objective

This study aimed to compare the fusion rate of polyetheretherketone (PEEK) and titanium-coated PEEK (Ti-PEEK) cages in transforaminal lumbar interbody fusion (TLIF).

Methods

The patient groups that underwent TLIF using PEEK and Ti-PEEK cages were identified and matched for age, sex, and whether the lumbar spine surgery was performed more than once using propensity scores. The rate of three-month postoperative vertebral endplate cyst sign (VECS), which was reported as a predictor of pseudoarthrosis at one year postoperatively, and the one-year and two-year postoperative fusions between the two groups were statistically compared.

Results

There were 34 patients (12 men and 22 women) in the PEEK group with a mean age of 69.8 ± 8.2 years and 36 intervertebral discs; there were 30 patients (11 men and 19 women) in the Ti-PEEK group with a mean age of 70.3 ± 9.6 years and 36 intervertebral discs. The operated levels were two discs (5.6%) in L2/3, four (11.1%) in L3/4, 21 (58.3%) in L4/5, and nine (25.0%) in L5/S in the PEEK group and were one (2.8%) in L3/4, 24 (66.7%) in L4/5, and 11 (30.6%) in L5/S in the Ti-PEEK group (P = 0.31).

The frequencies of positive VECS at three months postoperatively were four discs (11.1%) in the PEEK group and five (13.9%) in the Ti-PEEK group, with no significant difference (P = 0.72). Bone fusion rates at one year and two years postoperatively were 26 (72.2%) and 28 (77.8%), respectively, in the PEEK group, and 28 (77.8%) and 32 (88.9%), respectively, in the Ti-PEEK group, with no statistically significant difference (P = 0.59 at one year and P = 0.21 at two years). Among the five cases of positive VECS in the Ti-PEEK group, two cases (40%) had bone fusion at one year postoperatively around the cage but not through the cage.

Conclusion

There was no significant difference in the rate of bone fusion or VECS after TLIF between the PEEK and Ti-PEEK groups. Positive VECS is more appropriate for a finding of no bone fusion through the cage at one year postoperatively than for a finding that predicts pseudoarthrosis.

## Introduction

Lumbar spine fusion surgery is a common treatment for various degenerative diseases of the spine. The goal of this surgery is to stabilize the affected segment of the spine by fusing two or more vertebrae with bone grafts or metal implants. One of the major issues associated with the surgery is pseudoarthrosis. There are reportedly many factors that can affect the spinal fusion rate: patient health conditions (medical history, drug use, smoking, anemia, psychiatric disorders), patient preoperative spine-related factors (bone quality, diagnosis, spinal alignment, presence of infection), surgery-related factors (approach, minimally invasive (MIS) or not, type and size of instrumentation, bone graft, bone morphogenetic protein (BMP) use or not), surgeon-related factors (surgical skill and experience), and postoperative care (bracing, rehabilitation, behavioral restrictions, medications) [1].

Among these many factors, the cage material has received the most attention. Polyetheretherketone (PEEK) has an elastic modulus similar to that of cortical bone and radiolucency that makes evaluation of bone fusion relatively easy [2,3]. Titanium (Ti), on the other hand, has high biological affinity and is characterized by the possibility of surface processing that improves initial fixation [4].

A meta-analysis of fusion rates in posterior lumbar interbody fusion (PLIF) found that PEEK cages had inferior bone fusion rates compared to titanium cages [5]. On the other hand, another meta-analysis found no significant difference in fusion rates between PEEK and titanium cages in transforaminal lumbar interbody fusion (TLIF) and anterior cervical discectomy and fusion, with the titanium cage having a higher subsidence rate than the PEEK cage [6]. Therefore, the superiority of these two cage types is still controversial. While it is controversial which of the two biomaterials is better, the Ti-coated PEEK (Ti-PEEK) cage has become popular because it theoretically combines both of these advantages.

The primary objective of this study was to examine statistically whether Ti-PEEK cages improve bone fusion rate compared to uncoated PEEK cages, based on a review of prospectively accumulated data. The second objective was to evaluate the way bone fusion was achieved in Ti-PEEK cages examined based on computed tomographic (CT) images.

## Materials and methods

**Figure 1:**
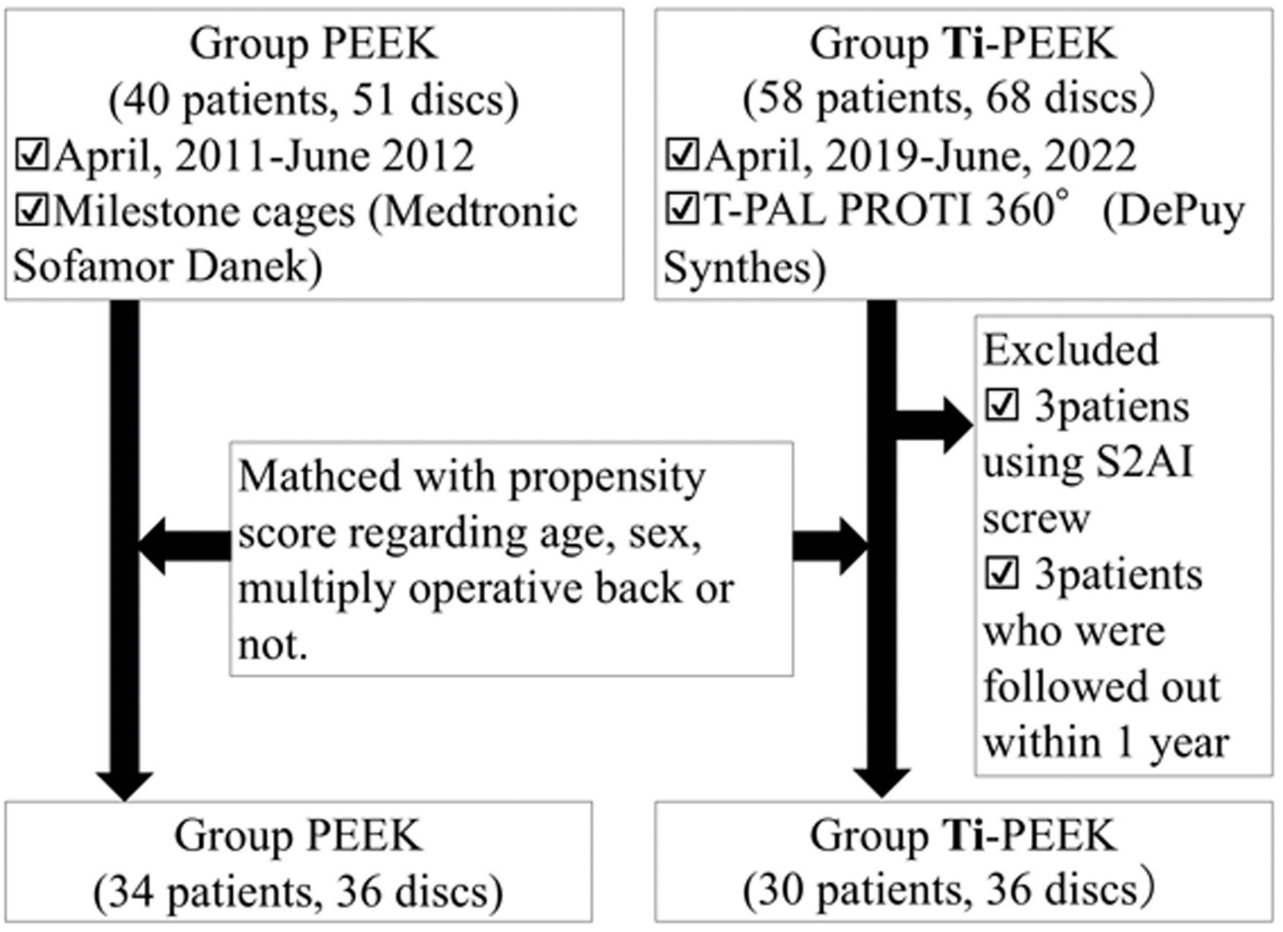
Extraction procedure for the two groups of patients.

This study was approved by the Ethics Committee of Shiga General Hospital in Moriyama, Japan. Patients were selected as shown in Figure 1. A total of 40 consecutive patients (15 men and 25 women; mean age 65.0 ± 12.7 years) with 51 intervertebral discs who underwent TLIF with PEEK cages for lumbar spinal canal stenosis and/or spondylolisthesis between April 2011 and June 2012 were identified. On the other hand, we identified 58 consecutive patients (28 men and 30 women) with 68 intervertebral discs that underwent one- or two-level TLIF for lumbar spinal canal stenosis and/or spondylolisthesis using Ti-PEEK cages from April 2019 to June 2022. From this group, three patients (three women) who were followed out and three patients (two men, one woman) who underwent fixation of more than three vertebrae using sacroiliac joint screws were excluded, and 52 patients with 62 intervertebral discs (26 men, 26 women; mean age 73.4 ± 9.2 years) were finally identified. Patients with sacroiliac screws were excluded because there were no cases of sacroiliac screw use in patients in the PEEK group, eliminating the possibility that the strong fixation force of the screw may have affected the rate of bone fusion. Although the centers where the two groups of patients underwent surgery were different, the corresponding author changed institutions and continued to perform the same surgical procedure on the two groups of patients, except for the type of cage. Propensity scores were calculated for the cages (not for the patients) in both groups for age, sex, and whether the discs had undergone multiple surgeries, and matched intervertebral discs from both groups were extracted based on these values. The reason for such matching was that there was a statistically significant difference (P < 0.01) by *t*-test in age between the two groups before matching, and the effect of postmenopausal osteoporosis could not be excluded in this study, which included elderly patients. In addition, multiple surgeries were reportedly an independent risk of pseudoarthrosis after TLIF in previous reports [7,8].

Surgery was performed in both groups by the open technique. Surgical procedures were standardized between the two groups. Morselized and block-shaped bone grafts from local bones were grafted in and around the cage, and a facet fusion technique was also performed on the opposite side of the entry in TLIF. If the local bone was insufficient, the iliac bone was harvested. Recombinant human bone morphogenetic protein-2 was not used in either group. All cages in the PEEK group were Milestone cages (Medtronic Sofamor Danek USA, Inc., Memphis, TN, USA), and all cages in the Ti-PEEK group were T-PAL PROTI 360°System (DePuy Synthes Spine, Raynham, MA, USA).

Radiological evaluation

**Figure 2:**
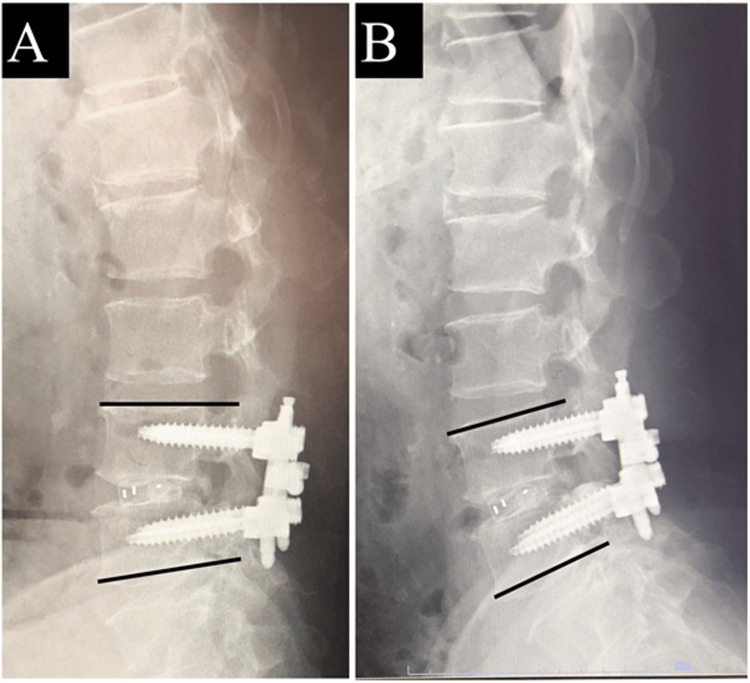
Cobb angle of the vertebral body endplate on the postoperative lateral radiographic image: (A) flexion, (B) extension.

Radiographic and multidetector-row CT images were taken in all the patients preoperatively, three months postoperatively, and one year postoperatively. In order to reduce radiation exposure, CT images were taken at two years postoperatively only in the patients in whom bone fusion could not be confirmed at one year postoperatively. Therefore, patients who were judged to have achieved bone fusion at one year postoperatively were judged to still have bone fusion at two years postoperatively, unless there were new abnormal findings on X-ray or clinical findings. Bone fusion was determined to have been achieved according to the past report [7] when the following conditions were all met: 1) Cobb angle of the vertebral body endplate did not change more than three degrees in flexion-extension on the postoperative lateral radiographic image (Figure 2). 2) There was no obvious gap between the vertebral endplate and the cage on CT images and no radiolucency around the pedicle screw on radiographic and CT images. 3) Bone bridging between the vertebral bodies through or around the cage on both sagittal and coronal views on CT images was seen.

**Figure 3:**
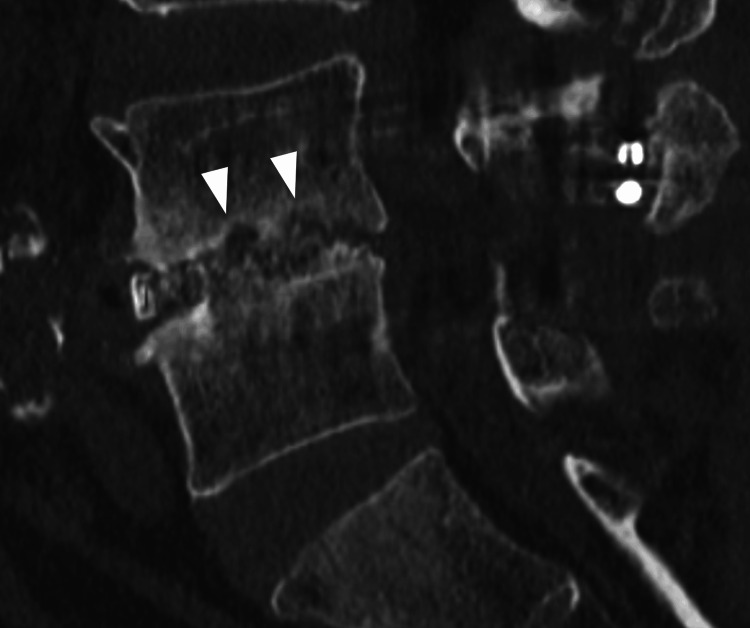
Vertebral endplate cyst sign on CT (arrow head).

Another radiological endpoint evaluated was vertebral endplate cyst sign (VECS) at three months postoperatively, which was first reported in 2012 as a predictive sign of pseudoarthrosis of lumbar interbody fusion at one year postoperatively and has been shown to be an imaging finding with high intra- and inter-rater reliability [7,8]. According to those reports, VECS is a circular or oval bony translucency seen near the upper and lower vertebral body endplates on sagittal and coronal CT images (Figure 3). Comparing between preoperative and three-month postoperative CT images, VECS positivity was defined as the occurrence of cyst sign postoperatively, or an increase in the size of a preoperative cyst if present. Two of the authors of this paper, blinded to each other, assessed the bone fusion and VECS. If they disagreed, they conferred and reassessed.

In cases where bone fusion was achieved using Ti-PEEK cages at one year postoperatively, the location of bone bridging (through or around the cage) was evaluated separately for VECS positive and negative at three months postoperatively.

Statistical analysis

EZR (version 1.68, Saitama Medical Center, Jichi Medical University, Saitama, Japan) was used for all the statistical analyses [9]. Age was compared by *t*-test. Propensity scores were calculated to extract the two groups of intervertebral discs as described before. The proportions of categorical variables between the two groups were compared using Fisher's exact probability test. Logistic regression analysis was used to compare bone fusion rates and frequency of VECS between the two groups. Sensitivity, specificity, and positive and negative predictive values of VECS as a predictor of pseudoarthrosis were calculated. P < 0.05 was considered statistically significant.

## Results

Demographic data of the two groups extracted by matching using propensity scores are summarized in Table 1. There were 34 patients in the PEEK group (12 men and 22 women) with a mean age of 69.8 ± 8.2 years and 36 intervertebral discs; there were 30 patients in the Ti-PEEK group (11 men and 19 women) with a mean age of 70.3 ± 9.6 years and 36 intervertebral discs. There were no significant differences in age, sex, and percentage of discs with multiple surgeries between the PEEK and Ti-PEEK groups. The operated levels in the PEEK group were two discs (5.6%) in L2/3, four (11.1%) in L3/4, 21 (58.3%) in L4/5, and nine (25.0%) in L5/S. The levels in the Ti-PEEK group were one disc (2.8%) in L3/4, 24 (66.7%) in L4/5, and 11 (30.6%) in L5/S. There was no statistically significant difference in the distribution of levels (P = 0.31).

**Table 1:** Demographic data of the two groups extracted by matching using propensity scores. P < 0.05 was considered statistically significant. #: P-value for the disc-level distribution.

	PEEK	Ti-PEEK	p
Patients (n)	34	30	N/A
Sex men/women	12 (35.3 %) / 22 (64.7 %)	11 (36.7 %) / 19 (63.3 %)	1
Age (years)	69.8 ± 8.2	70.3 ± 9.6	0.822
Discs	36 discs	36 discs	N/A
L2/3	2 (5.6%)	0 (0%)	-
L3/4	4 (11.1%)	1 (2.8%)	-
L4/5	21 (58.3%)	24 (66.7%)	-
L5/S	9 (25.0%)	11 (30.6%)	0.31 (#)
Multiply operated disc	4 (11.1%)	4 (11.1%)	1

The rate of bone fusion and VECS are summarized in Table 2. The frequencies of VECS positive at three months postoperatively were four (11.1%) in the PEEK group and five (13.9%) in the Ti-EEKP group, with no statistically significant difference (P = 0.72, odds ratio = 1.29 (95% CI: 0.32-5.26)). Bone fusion rates at one year and two years postoperatively were 26 (72.2%) and 28 (77.8%), respectively, in the PEEK group and 28 (77.8%) and 32 (88.9%), respectively, in the Ti-PEEK group, with no statistically significant difference (P = 0.59 at one year and P = 0.21 at two years).

**Table 2:** Rate of vertebral endplate cyst sign at three months postoperatively and bone fusion at one year and two years postoperatively. P < 0.05 was considered statistically significant.

	PEEK	Ti-PEEK	P
Vertebral endplate cyst sign positive	4 (11.1%)	5 (13.9%)	0.72 (odds ratio = 1.29 (95% CI: 0.32–5.26))
Fusion rate at one year	26 (72.2%)	28 (77.8%)	0.59 (odds ratio = 1.35 (95% CI: 0.46–3.93))
Fusion rate at two years	28 (77.8%)	32 (88.9%)	0.21 (odds ratio = 2.29 (95% CI: 0.62–8.41))

The relationship between positive VECS at three months postoperatively and bone fusion at one year postoperatively in the PEEK group and the Ti-PEEK group are summarized in Table 3. In the PEEK group, no cases with positive VECS at three months lead to bone fusion at one year postoperatively. By contrast, in the Ti-PEEK group, two (40%) of the five VECS-positive cases had bone fusion at one year. In the PEEK group, the sensitivity, specificity, and positive and negative predictive values of VECS as a predictor of pseudoarthrosis at one year were 40%, 100%, 100%, and 81.3%, respectively. In the Ti-PEEK group, the sensitivity, specificity, and positive and negative predictive values of VECS were 37.5%, 92.9%, 60%, and 83.9%, respectively.

**Table 3:** Relationship between the vertebral endplate cyst sign (VECS) at three months postoperatively and bone fusion at one year postoperatively in the PEEK and Ti-PEEK groups. P < 0.05 was considered statistically significant. VECS: vertebral endplate cyst sign, PEEK: polyetheretherketone, Ti-PEEK: titanium-coated polyetheretherketone

PEEK	Bone fusion (-)	Bone fusion (+)	Total
Positive VECS	4 (true positive)	0 (false positive)	4
Negative VECS	6 (false negative)	26 (true negative)	32
Total	10	26	36
Ti-PEEK	Bone fusion (-)	Bone fusion (+)	Total
Positive VECS	3 (true positive)	2 (false positive)	5
Negative VECS	5 (false negative)	26 (true negative)	31
Total	8	28	36

**Figure 4:**
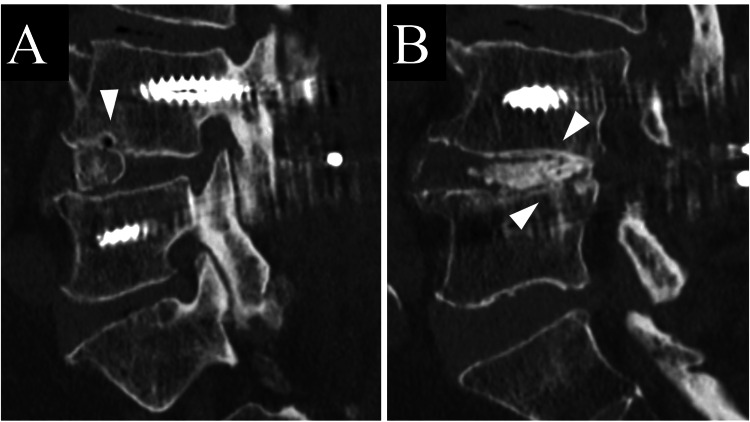
The VECS-positive cases had bone fusion around the cage but not through the cage. (A) Vertebral endplate cyst sign (VECS) at three months postoperatively (arrowhead). (B) Bone bridging around the cage at one year postoperatively (arrowhead).

Among all the cases that achieved bone fusion using Ti-PEEK cages at one year postoperatively (n = 28), all VECS-negative cases (n = 26) had bone bridging both through and around the cage, while all of the VECS-positive cases (n = 2) had bone fusion around the cage but not through the cage (Table 4, Figure 4).

**Table 4:** Rlationship between the location of bone bridging and the results of the vertebral endplate cyst sign in cases where bone fusion was achieved at one year postoperatively with Ti-PEEK. VECS: vertebral endplate cyst sign, PEEK: polyetheretherketone

	Fusion achieved with VECS-negative cases (n = 26)	Fusion achieved with VECS-positive cases (n = 2)
Bone bridging through the cage	26 (100%)	0 (0%)
Bone bridging around the cage	26 (100%)	2 (100%)

## Discussion

Bone fusion is one of the most important outcomes of spinal fusion surgery. It has been reported that pseudoarthrosis can lead to persistent or recurrent pain in the back or legs, reduced spinal stability and mobility, hardware failure or loosening, infection or inflammation, or nerve damage or compression [10].

The superiority of Ti-PEEK cages over PEEK cages is still controversial. In a prospective, randomized study, Rickert et al. reported that the three-month postoperative bone fusion rate after TLIF at the L2-L5 level between one and two vertebrae was 91.7% both in two groups, with no statistically significant difference [11]. Vanek et al. reported in a single-center, prospective, randomized study that in TLIF at the L3-S levels, in which the two-year postoperative bone fusion rates for PEEK and Ti-PEEK cages were 85.4% and 92.5%, respectively, with no statistically significant difference [12]. In a prospective, randomized study, Schnake et al. found that bone growth through the cage was observed in both PEEK and Ti-PEEK cages at one year postoperatively in PLIF at 100%, with good radiological evaluation results in both groups and no statistical difference [13].

By contrast, some reports indicated the possible superiority of Ti-PEEK over PEEK cages. Kashii et al. used PEEK and Ti-PEEK cages in the same intervertebral space in PLIF and performed CT imaging evaluation at three months postoperatively. They reported that vertebral cancellous condensation around the Ti-PEEK cage was more common than that in the PEEK cage, suggesting bone ingrowth on the cage surface, and that the Ti-PEEK cage may cause solid fusion [14]. Singhatanadgige et al. reported that in a randomized study in MIS-TLIF, the six-month postoperative bone fusion rate was 91.8% for Ti-PEEK and 76% for PEEK, which was significantly higher, but there was no difference at 12 months postoperatively [15]. In a prospective randomized study, Willems et al. found that the bone fusion rate of PLIF was significantly higher at one year postoperatively in Ti-PEEK cages (93.9%) than in PEEK cages (65.6%) [16]. In a prospective randomized multicenter study, Hasegawa et al. found that Ti-PEEK showed better fusion than PEEK at six months postoperatively in PLIF, but there was no statistically significant difference at 12 months [17]. As far as we investigated, the advantage of Ti-PEEK cages over PEEK cages may be seen in the relatively early postoperative period, but there is no significant difference in bone fusion rate between the two groups after one year postoperatively.

The results of the present study, especially the bone fusion rate of 77.8% in the PEEK group and 88.9% in the Ti-PEEK group at two years postoperatively (P = 0.21), could be interpreted as a result of insufficient statistical power due to the small numbers, even though Ti-PEEK actually has a better bone fusion rate than PEEK. However, the data on bone fusion in the present study are particularly similar to those of Vanek and colleagues [12] described earlier, where the bone fusion rate two years after surgery only tended to be higher in the Ti-PEEK group than that in the PEEK group with no statistical significance. Therefore, we conclude that our data do not support the superiority of Ti-PEEK over PEEK. At least, the present study and the previous reports suggest that Ti-PEEK cages have not performed as well clinically as expected when they became commercially available. The reason that Ti coating does not provide an obvious improvement in bone fusion rate is presumably due to the wide variety of factors mentioned in the introduction. In addition, another concern with Ti coating is wear and delamination of the coating that occurs during the impaction of the titanium-coated cage [18]. It has been pointed out that wear of the Ti coating may induce a localized adverse inflammatory reaction [19,20], although the effect of wear on clinical outcomes, including bone fusion, is not yet clear, and further investigation is needed.

Regarding the relationship between bone fusion at one year postoperatively and VECS at three months postoperatively, the positive predictive value of positive VECS as a predictor of pseudoarthrosis in Ti-PEEK was not 100%, unlike PEEK or titanium cages in the previous report [8]. However, in Ti-PEEK, when the VECS was positive, even when bone fusion was achieved, bone bridging was achieved around the cage, not through the cage. This suggests that the positive VECS is a finding indicative of cage micromotion, as pointed out in the previous report [8]. On the other hand, even if the cage becomes unstable postoperatively, bone fusion may still be achieved around the cage, suggesting the importance of additional bone grafting techniques and block-shaped bone graft and morselized bone behind the cage.

The present study has several limitations. First, the shape and size of the PEEK cage are different from that of the Ti-PEEK cage. Therefore, the effects of the Ti coating are not strictly comparable. Second, the timing of the surgery differs between the two groups, and the amount of grafted bone and the pedicle screw system are not standardized. Third, the sample size of the two groups is small. Although it is necessary to increase the number in the form of a multicenter study, this study focused more on standardizing the surgical technique. Fourth, clinical outcomes assessed by self-reported outcomes including the Oswestry Disability Index and the visual analogue scale for pain and numbness were only available for the Ti-PEEK group. Therefore, the clinical outcomes could not be compared between the two groups. Finally, we have not been able to take into account the many other factors mentioned above that can affect the bone fusion rate. Future studies on a very large scale are needed to comprehensively evaluate these factors.

## Conclusions

The bone fusion rates of TLIF were statistically compared between PEEK cages and Ti-PEEK cages under the same surgical technique conditions. When matched for age, sex, and whether the lumbar spine surgery was performed more than once using propensity scores, there was no significant difference in the rate of bone fusion at one and two years postoperatively or VECS at three months postoperatively after TLIF between the PEEK and Ti-PEEK groups. The present study suggested that Ti-PEEK cages have not performed as well clinically as expected when they became commercially available, although there is a problem of the small number of subjects in this study. In addition, the transition between pre- and post-operative CT imaging findings showed that VECS is more appropriate for a finding of no bone fusion through the cage than for a finding that predicts pseudoarthrosis. Future large-scale studies are needed to comprehensively examine the various factors affecting bone fusion.
